# Polymorphism of rs1836882 in *NOX4* Gene Modifies Associations between Dietary Caloric Intake and ROS Levels in Peripheral Blood Mononuclear Cells

**DOI:** 10.1371/journal.pone.0085660

**Published:** 2013-12-31

**Authors:** Qiang Liu, Hong Li, Ningfu Wang, Huaihong Chen, Qihui Jin, Ruoyu Zhang, Jing Wang, Ying Chen

**Affiliations:** 1 Department of Gerontology, the Second Affiliated Hospital, Zhejiang University School of Medicine, Hangzhou, China; 2 Department of Cardiology, the Affiliated Hangzhou Hospital, Nanjing Medical University, Hangzhou, China; Hokkaido University, Japan

## Abstract

Excessive caloric intake is a contributing risk factor for human metabolic disorders. Caloric restriction may prolong a person’s life by lowering the incidence of deadly diseases. Reactive oxygen species (ROS) in peripheral blood mononuclear cells (PBMC) have been associated with the biochemical basis of the relationship between caloric intake and pathophysiologic processes. Polymorphisms associated with ROS generation genes are being increasingly implicated in inter-individual responses to daily caloric intake alterations. In the current study, a single nucleotide polymorphism, rs1836882, in the nicotinamide adenine dinucleotide phosphate oxidase 4 (*NOX4*) gene’s promoter region was found to modulate associations between dietary caloric intake and ROS levels in PBMC. Based on rs1836882, 656 Chinese Han participants were classified into CC, CT and TT genotypes. ROS levels in PBMC were significantly higher in the CC or CT genotypes compared with the TT genotype with the same increases in daily caloric intake. Using an electrophoretic mobility shift assay, *NOX4* promoter region with rs1836882 (T) was observed to have a higher affinity for hepatocyte nuclear factor gamma (HNF3γ) protein than rs1836882 (C). HNF3γ protein over-expression decreased *NOX4* gene transcriptional activity in the TT genotype more than in the CC genotype (5.68% *vs.* 2.12%, *P*<0.05) in a dual luciferase reporter assay. By silencing the *NOX4* gene using small interfering RNA or over-expressing HNF3γ using an expression plasmid, serum from high dietary caloric intake participants decreased ROS levels in PBMC of the TT genotype more than in the CC or CT genotype via HNF3γ down-regulating the *NOX4* gene expression signaling pathway. This is the first study to report on the functions of phenotypes of rs1836882 in the *NOX4* gene, and it suggests rs1836882 as a candidate gene for interpreting inter-individual ROS levels differences in PBMC induced by alterations in daily caloric intake.

## Introduction

Excessive energy intake can cause detrimental effects on the body, and is often related to disease risk factors because of associations between metabolic disorders and the probability of disease [1-3]. It is widely believed that caloric restriction lowers the incidence of several kinds of diseases, such as cancer, diabetes, atherosclerosis, cardiovascular disease, and neurodegenerative diseases [4,5]. 

The mechanisms responsible for the effects of caloric restriction on the pathogenesis of many diseases are not entirely clear. The free radical theory is one of the most accepted theories explaining the biochemical basis for associations between caloric restriction and its beneficial effects [6-8]. Studies suggest that caloric restriction may significantly decrease the rate of mitochondrial reactive oxygen species (ROS) generation and damage to macromolecules, including mitochondrial DNA, in organs of calorie restricted animals [9,10]. Decreases in mitochondrial ROS generation have been reported to be localized at complex I in the electron transport chain, where NADH directly feeds electrons into this complex [11]. Besides mitochondrial ROS those receive electrons from NADH, caloric restriction has been reported to decrease the generation of intracellular ROS receiving electrons from NADPH, especially in the cardiovascular system [12]. Mice on a calorie-restricted diet (beginning at 14 weeks of age and continuing throughout their life) showed recovered endothelial vasodilation by blunting age-related increases in NADPH oxidase activity, p67 expression and oxidative stress in the arteries [13]. A short-term caloric restriction (for 8 weeks) was also shown to reduce vascular oxidative stress via reduced NADPH oxidase-mediated superoxide production [14]. Similar results were obtained after 3 months of caloric restriction in older rats [15]. Ketonen et al. reported that caloric restriction reversed obesity-induced vascular oxidative stress, partly by diminishing superoxide production from NADPH [16].

NADPH oxidases are the only known enzyme family with the sole function of producing ROS. In the origination of ROS from NADPH, NADPH oxidase is the key component for providing electrons to oxygen. Of the catalytic NADPH oxidase subunits (NOX), NOX4 (Entrez Gene: 50507) is the most widely distributed isoform [17]. To date, the *NOX4* gene has been reported to be involved in multiple pathogeneses, including cell senescence [18], apoptosis [19], endothelial dysfunction [20], angiogenesis [21], atherosclerosis and vascular aging [22], cardiac remodeling [23], and neoplasms [24]. A review by Altenhofer et al. [17] suggests that the *NOX4* gene may serve as a potential therapeutic target for indications of disease, including stroke and heart failure [25,26].

In our previous study, aided by the research from Panowski et al. [27], we reported that through the promotion of hepatocyte nuclear factor gamma (HNF3γ; Entrez Gene: 3171) protein binding to the *NOX4* gene promoter region and inhibiting *NOX4* gene expression, caloric restriction can decrease production of intracellular ROS and suppress endothelial cell senescence [28]. After further analyzing the *NOX4* promoter region, we found a single nucleotide polymorphism (SNP), rs1836882, located near one of the HNF3γ binding sites. This encouraged us to investigate whether rs1836882 is able to affect the binding of HNF3γ to the *NOX4* promoter region and consequently alter transcriptional *NOX4* gene activity responding to dietary caloric intake changes.

Peripheral blood mononuclear cells (PBMC) are a type of peripheral blood cell that continuously interact between blood cells and the entire body, and may change their intracellular oxidative stress-associated gene expression in response to dietary caloric intake [29]. PBMC have been reported to contain a multitude of distinct multi-potent progenitor cell populations and possess the potential to differentiate into various kinds of cells under appropriate conditions [30]. Increased ROS levels from NADPH oxidases in PBMC has been associated with several pathogeneses, including chronic obstructive pulmonary disease [31], type 2 diabetes [32], coagulation activation [33], endothelial dysfunction [34] and hypertension [35]. Therefore, the formation of ROS and oxidative stress in PBMC may be one of mechanisms linking caloric restriction and its possible effects on human health.

There are varying inter-individual responses to diet intervention [36,37]. It is possible that these differences depend on genetic variation in metabolic-sensitive genes. In this regard, investigating whether and how polymorphism of rs1836882 modulates associations between dietary caloric intake and ROS levels in PBMC may help further clarify the mechanisms behind individual differences seen with diet intervention.

This study investigated: (1) if there is any association between dietary caloric intake and ROS levels in PBMC from healthy Chinese Han people; (2) if polymorphism of rs1836882 is able to modify this association; and (3) if HNF3γ binding-induced *NOX4* gene transcriptional activity alteration is partly responsible for this modulation.

The results suggest that polymorphism of rs1836882 is able to modulate associations between dietary caloric intake and ROS levels in PBMC; and HNF3γ down-regulating the *NOX4* gene expression signal pathway partly accounts for this modulatory effect.

## Materials and Methods

### Participants and ethical considerations

A total of 656 unrelated Han Chinese healthy volunteers aged 25–45 years were selected for the current study from 1543 people attending health examinations and living in Hang Zhou City, China. All participants were non-smokers, normal weight (18.5 kg/m^2^<BMI<24.9 kg/m^2^), of good general health and free of diseases or conditions related to oxidative stress (cancer, diabetes, heart disease, impaired renal function, Alzheimer’s disease, stroke, asthma, and endocrine disorders), and were not taking any medicines or supplements, such as vitamin A. Other exclusion criteria included anorexia or bulimia nervosa, a large weight change (>5% of body weight) in the past 6 months, inability to fast for 6 h, pregnancy, non-Han race people (by self-identification), and declining to sign informed consent for genetic studies. 

The study was conducted according to the principles of the Declaration of Helsinki and was approved by the Hangzhou First People’s Hospital Ethics Committee. Written informed consent was obtained from all participants.

### Phenotype measurements

Height, weight, systolic blood pressure, and diastolic blood pressure were measured for each participant. BMI was calculated as weight (kg) divided by height squared (m^2^). All participants fasted for 12 h before blood collection. Plasma glucose, serum lipid concentrations (total cholesterol, HDL, LDL and triglycerides) were measured with the appropriate Roche Diagnostics reagents. Based on the procedure adopted by Vaccaro et al. [38], dietary habits were investigated with the use of a 64-item semi-quantitative food frequency questionnaire administered by trained dietitians and designed on the basis of previous validity and reliability in Chinese population studies [39]. Total energy expenditure was calculated from activity patterns, including basal metabolic rate (using the Harris-Benedict equation), physical activity in 24 h, and the specific dynamic action of food.

### SNP selection and genotyping

SNP in the promoter regions (-2000 bp to +100 bp) of *NOX4* genes were searched using the dbSNP database (www.ncbi.nlm.nih.gov/SNP, Build 137 for Human). SNP with unknown heterozygosity, minor allele frequency below 10%, and monomorphic/unknown genotype in Asians were excluded. SNP of rs1836882 (C/T) were selected and analyzed. Genotypes were determined by direct sequencing (Invitrogen, Shanghai, China). Sequencing results were compared with the reference human sequence (Homo sapien chromosome 11, GRCh37, gi224589802:c89234364-89231364).

### Isolation and primary culture of PBMC

PBMC from all participants were isolated from 20 ml of heparinized peripheral blood using Ficoll-Paque (Invitrogen) gradient centrifugation at 500*g* for 20 min and washed twice with phosphate buffered saline (pH 7.2). Washed PBMC were either stored at -70°C before further use or cultivated in an RPMI-1640 culture medium (Invitrogen) containing 10% fetal bovine serum in 6-well culture plates at 37°C, 95% O_2_, and 5% CO_2_ before further gene transfection and serum treatment.

### ROS detection in PBMC

ROS in PBMC from all participants was measured using a dichloro-dihydro-fluorescein diacetate assay (DCFH-DA). The intensity of fluorescence was analyzed in a flow cytometer. Results are expressed as the average intensity fluorescence in the analyzed cells.

### Electrophoretic mobility shift assay

Nuclear proteins from freshly prepared PBMC were extracted using a nuclear extraction kit (Pierce, Rockford, IL, USA). EMSA were performed using gel shift assay systems (Promega Biotech, Beijing, China) with a biotinylated probe under the guidelines provided. Competition experiments were performed using a 50-fold molar excess of unlabeled probe. Biotinylated fragments were detected with a Light Shift Chemiluminescent EMSA kit (Pierce) according to the manufacturer’s instructions. (For details see Text S1).

### Construction of plasmids

Lucifer’s reporter gene was performed to analyze the effect of rs1836882 (C/T) on *NOX4* gene transcriptional activity. Two kinds of luciferase expression plasmids were constructed using genomic fragments amplified by PCR from individuals homozygous for C-C or T-T in rs1836882: pGL3-basic-NOX4-(C) and pGL3-basic- NOX4-(T). In addition, the fragment containing the open reading frame of the *HNF3γ* gene was ligated into the pcDNA3.1 vector (Invitrogen), and the recombinant expression plasmid (named pcDNA3.1-HNF3γ) was constructed. (For details see Text S1).

### Transfection of HNF3γ plasmids for PBMC or HEK293 cells

HNF3γ plasmid transfection was performed using Lipofectamine 2000 (Invitrogen). On reaching 60% confluence, PBMC or HEK293 cells were incubated with the HNF3γ plasmid-Lipofectamine 2000 complexes at 37°C for 4 h, followed by recovery in growth RPMI-1640 medium for PBMC or MEM medium for HEK293. Cells were used in experiments 72 h after transfection.

### Transfections and dual luciferase reporter assay

HEK293 cells were seeded into 48-well plates at a density of 1×10^4^ cells/well prior to transfection. Transfection was performed using Lipofectamine 2000 (Invitrogen) according to the manufacturer’s instructions. In the absence or presence of 100 ng pcDNA3.1-HNF3γ vector, cells were co-transfected with 150 ng of preconstructed pGL3-basic-NOX4 (C or T) luciferase reporter plasmid and 1.0 ng Renilla reporter control plasmid pRL-TK (Promega) for each well. Luciferase activity was measured 24 h later using the Dual-Luciferase Reporter Assay System (Promega) on a luminometer (Promega) according to the manufacturer’s instructions. Results were normalized to Renilla luciferase activity and data expressed as relative luciferase activity.

### Transfection of *NOX4* and *HNF3γ* siRNA for PBMC

*NOX4* or *HNF3γ* siRNA transfection was performed using Lipofectamine 2000 (Invitrogen). Brieﬂy, transfection was performed on 80% conﬂuent cells with Lipofectamine 2000 reagent diluted in RPMI-1640 medium according to the manufacturer’s instructions. Cells were incubated with siRNA for 4 h at 37°C and then siRNA-EMG-2 medium was removed and replaced with growth medium. Cells were used in experiments 48 h after transfection. (For details see Text S1).

### Serum treatment for PBMC

To investigate modulation of rs1836882 on the correlation between caloric intake and PBMC ROS levels *in vitro*, the 656 participants were classified into three groups (low, middle and high) based on their caloric intake levels. PBMC with rs1836882 gene types CC, CT or TT were randomly isolated from participants in the low group. Serum from the high caloric intake group (SHC) and serum from the low caloric intake group (SLC) were of type AB and were collected as described previously [40].

PBMC were grown in RPMI-1640 culture medium (Invitrogen) containing 10% fetal bovine serum until serum treatment. For serum treatment, after a 24-h starvation period with serum-free RPMI-1640 medium, PBMC with genotypes CC, CT or TT were treated for 24 h in the following conditions: (1) cells were cultured in RPMI-1640 medium with SLC (10%) (Control group); (2) cells were cultured in RPMI-1640 medium with SHC (10 %) (SHC group); (3) cells were pretransfected with *NOX4* siRNA and cultured in 10% SLC RPMI-1640 (NOX4 group); (4) cells were pretransfected with *NOX4* siRNA and cultured in 10% SHC RPMI-1640 (SHC+NOX4 group); (5) cells were pretransfected with *HNF3γ* siRNA and cultured in 10% SLC RPMI-1640 (HNF3γ group); (6) cells were pretransfected with *HNF3γ* siRNA and cultured in 10% SHC RPMI-1640 (SHC+HNF3γ group); (7) cells were pretransfected with HNF3γ over-expression plasmid and cultured in 10% SHC RPMI-1640 (HNF3γ over-expression group); and (8) cells were pretransfected with HNF3γ plasmid and cultured in 10% SHC RPMI-1640 (SHC+HNF3γ over-expression group). 

### Real-time RT-PCR analysis

Total cellular RNA from PBMC was prepared using Trizol (Invitrogen). The OD260/OD280 ratio for all samples was between 1.8 and 2.0. After quantification, 1 μg of total cellular RNA was used for reverse transcription with a RT-PCR kit (Invitrogen) and an oligo (dT) primer. PCR was conducted in a 50 μL reaction system containing 200 nmol/L of primer (see Text S1), 120 nmol/L TaqMan probe and premix Ex Taq™ (Takara Biotechnology (Dalian), China). Samples were amplified in the Applied Biosystems 7900 HT Fast Real-Time PCR System (Applied Biosystems, Foster City, CA, USA) for 40 cycles at the following conditions: denaturation for 10 s at 95°C, and annealing and extension for 40 s at 60°C. Relative mRNA expression levels of *NOX4* or *HNF3γ* were calculated using the Δ Δ Ct method, and β-actin was regarded as the internal control. (For details see Text S1).

### Western blotting

PBMC were lysed and protein concentrations were determined by the Bradford assay (Sigma-Aldrich, St. Louis, Missouri, USA). Total proteins were separated by 15% SDS-PAGE before being transferred to nitrocellulose membranes. Membranes were blocked with 5% non-fat milk. Rabbit polyclonal antibodies for human NOX4 and HNF3γ (Santa Cruz Biotechnology, Inc, Santa Cruz, CA, USA) was used at a dilution of 1:100. Rabbit polyclonal anti-β-actin antibodies were used as a protein loading control (1:1000; Sigma). Anti-rabbit HRP-conjugated NOX4 or HNF3γ secondary antibodies (Santa Cruz) were diluted 1:1000 and visualized using a chemiluminescent kit (Pierce).

### Statistical analysis

Data are presented as mean ± SD unless otherwise stated. Hardy-Weinberg equilibrium, linkage disequilibrium, and haplotype frequencies were determined and estimated using the SHEsis online program (http://analysis2.bio-x.cn/myAnalysis.php) [41]. SPSS Statistics version 17.0 (SPSS Inc., Chicago, IL, USA) was also used. General linear model ANOVA was used to test genotype effects. Each variable was examined for normal distribution, and skewed variables were tested after Box-Cox transformation using Mintab software (Minitab Inc., State College, PA, USA). A *P*-value less than 0.05 was considered statistically signiﬁcant.

## Results

### Distribution of *NOX4* gene promoter region SNP in the study population

The frequency of the minor allele C in the 656 participants was 0.362, which was similar to the frequency in the Chinese Han population (0.344; http://www.hapmap.org/). The frequencies of the CC, CT, and TT genotypes of rs1836882 among the 656 Chinese Han participants were 13.7%, 45.0%, and 41.3%, respectively. The genotype distribution did not deviate from Hardy-Weinberg equilibrium (*P*=0.497).

### Clinical, metabolic and dietary characteristics in the study population

As shown in Table 1, the clinical, metabolic and dietary characteristics did not differ among genotypes according to polymorphism of rs1836882. Current smoking and alcohol drinking status showed no differences among the three genotypes.

**Table 1:** Clinical, metabolic and dietary characteristics of the study population.

	CC	CT	TT	*P* value
	(n=90)	(n=295)	(n=271)	genotype
Sex (male: female)	41:49	139:156	125:146	0.955
Age (years)	39.36±6.67	39.66.33±6.17	38.83±6.73	0.308
Drinkers (%)	24.1	27.1	24.7	0.497
SBP (mmHg)	124.86±7.90	123.06±9.02	124.68±9.22	0.061
DBP (mmHg)	74.11±7.21	72.97±7.11	73.19±7.03	0.411
BMI (kg/m^2^)	22.66±1.52	22.84±1.41	24.74±1.40	0.474
Wc(cm)	75.80±8.34	75.83±8.47	76.54±8.77	0.568
Glucose (mmol/L)	5.14±0.55	5.14±0.57	5.08±0.59	0.461
Triglycerides (mmol/L)	1.21±0.41	1.15±0.38	1.16±0.37	0.495
Total cholesterol (mg/dl)	4.20±0.64	4.21±0.70	4.29±0.70	0.327
LDL cholesterol (mg/dl)	2.34±0.59	2.33±0.63	2.39±0.64	0.567
HDL cholesterol (mg/dl)	1.29±0.20	1.25±0.20	1.27±0.19	0.438
FFA (mmol/L)	0.69±0.27	0.74±0.37	0.71±0.43	0.469
Homocysteine (μmol/l)	14.32±1.44	14.26±2.18	13.99±2.05	0.208
Total energy expenditure (kcal)	2182.27±393.29	2209.04±404.68	2170.63±429.79	0.536
Estimates of daily nutrient intake				
Total energy intake (kcal/d)	2361.50±173.53	2322.51±195.99	2328.31±196.37	0.241
Carbohydrate (% of energy intake)	52.38±3.96	52.66±6.09	52.78±4.78	0.824
Protein (% of energy intake)	18.67±1.83	18.33±2.08	18.59±2.32	0.264
Fat (% of energy intake)	30.05±2.65	30.37±3.77	31.14±4.89	0.713
Crude ﬁber (g)	10.91±2.29	11.15±2.37	11.12±2.38	0.699
PUFA/SFA	1.46±0.08	1.45±0.10	1.45±0.09	0.641

### ROS levels, *NOX4* and *HNF3γ* gene expression in PBMC of study population

As shown in Table 2, the polymorphism was associated with differences in ROS levels, and *NOX4* and *HNF3γ* gene expression in PBMC (all *P*<0.01). The CC genotype had significantly higher ROS levels, *NOX4* mRNA and NOX4 protein levels (all *P*<0.01) *vs.* the other two genotypes (CT and TT). HNF3γ gene expression in PBMC showed no significant differences among the three genotypes.

**Table 2:** Cytokine, oxidative stress and vascular endothelial function in the study population.

	CC	CT	TT	*P* value
	(n=90)	(n=295)	(n=271)	genotype
ROS in PBMC	49.07±20.90	35.83±12.52*	29.98±11.12* **^*#*^**	*P* < 0.01
NOX4 mRNA in PBMC	0.91±0.31	0.52±0.15*	0.51±0.16*	*P* < 0.01
NOX4 protein in PBMC	0.49±0.23	0.25±0.11*	0.24±0.12*	*P* < 0.01
HNF3γ mRNA in PBMC	0.74±0.20	0.70±0.23	0.72±0.23	0.306
HNF3γ protein in PBMC	0.59±0.15	0.60±0.16	0.60±0.16	0.925

* *P*<0.05 *vs*. CC group; ***^#^*** *P*<0.05 *vs*. CT group.

### Relationship between energy intake per day and ROS levels in PBMC

**Figure 1:**
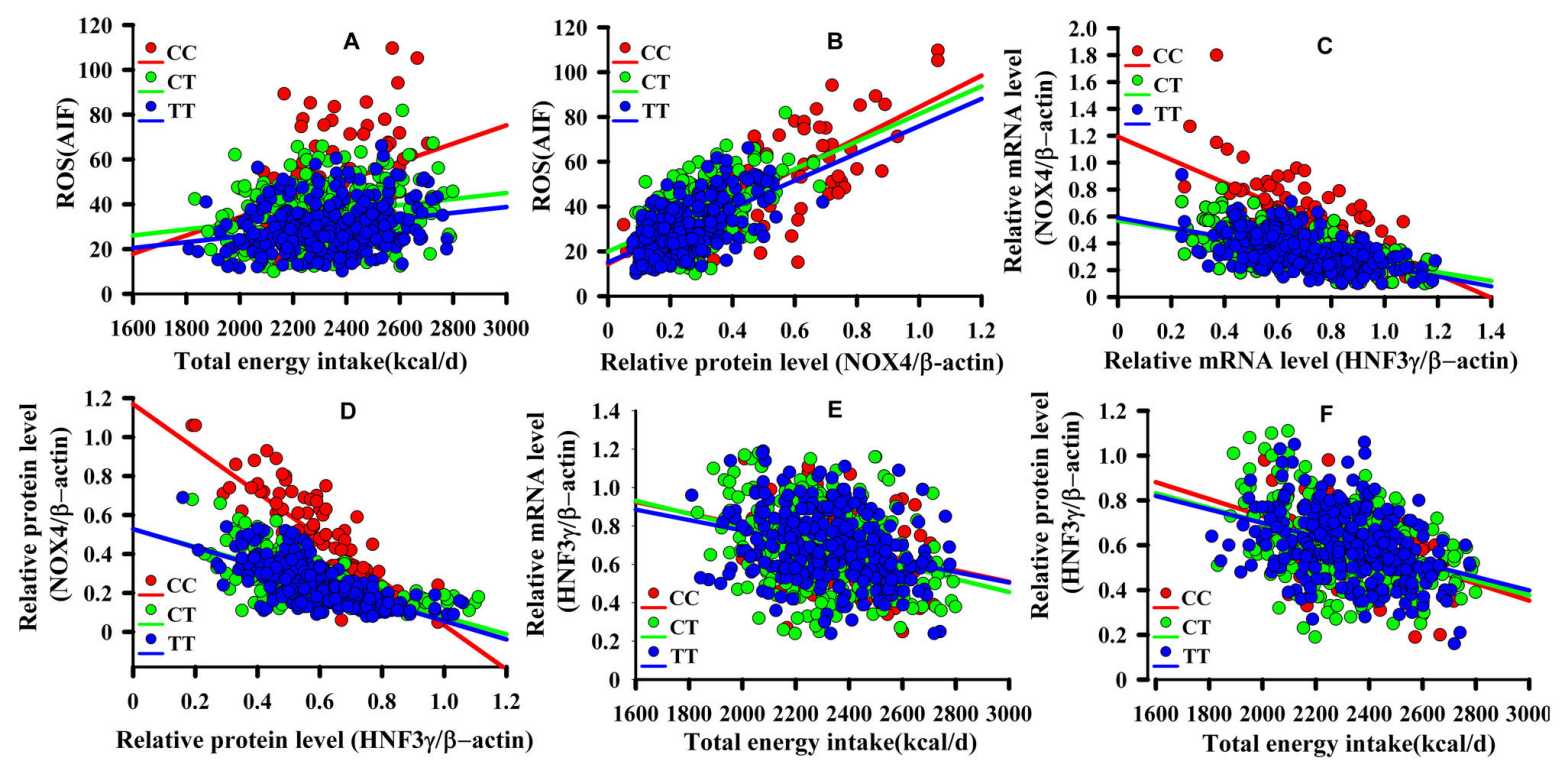
Effects of rs1836882 polymorphism on correlations between caloric intake, ROS levels, and NOX4 and *HNF3γ* gene expression. Regression lines for every genotype are indicated in A–F. A: Correlation between energy intake and ROS levels in PBMC among the three genotypes (*P* for interaction=0.005); B: Correlation between NOX4 protein levels and ROS levels in PBMC among the three genotypes (*P* for interaction=0.234); C: Correlation between *HNF3γ* and NOX4 mRNA levels in PBMC among the three genotypes (*P* for interaction=0.032); D: Correlation between HNF3γ and NOX4 protein levels in PBMC among the three genotypes (*P* for interaction=0.008); E: Correlation between energy intake per day and *HNF3γ* mRNA levels in PBMC among the three genotypes (*P* for interaction=0.516); and F: Correlation between energy intake per day and HNF3γ protein levels in PBMC among the three genotypes (*P* for interaction=0.740). AIF: average fluorescence intensity; ROS: reactive oxygen species; PBMC: peripheral blood mononuclear cells.

Correlations between energy intake and ROS levels in PBMC were examined among the three genotypes. A significant positive correlation was observed in all three genotypes between ROS levels in PBMC and total energy intake per day. However, the slopes from the three genotypes were significantly different (slop=0.041, 0.014 and 0.013 for CC, CT and TT genotypes, respectively; all *P*<0.05) for genotype and energy intake interaction effects, which caused a nearly double increase in PBMC ROS levels in CC genotypes *vs.* CT or TT genotypes with the same total energy intake level (Figure 1A).

### Relationship between ROS levels and *NOX4* gene expression in PBMC

Correlations between NOX4 protein levels and ROS levels in PBMC were examined among the CC, CT and TT genotypes. There were no significant differences in slope among the three genotypes (*P*=0.234) for NOX4 protein levels and genotype interaction effects. A positive correlation between NOX4 protein levels and ROS levels in PBMC was observed for all three genotypes (*r*=0.752, 0.535 and 0.604 for CC, CT and TT genotypes, respectively; all *P*<0.05) (Figure 1B).

### Relationship between *HNF3γ* and *NOX4* gene expression in PBMC

A significant negative association between *HNF3γ* and *NOX4* gene expression was observed in all three genotypes (Figure 1C and 1D). However, the slopes of the three genotypes showed a significant difference (*P*<0.05) for interaction between HNF3γ expression and genotype in both mRNA and protein levels. A one-unit decrease in *HNF3γ* mRNA levels resulted in 0.783, 0.099 and 0.01 unit increases in *NOX4* mRNA levels in CC, CT and TT genotypes, respectively. Similarly, a one-unit decrease in HNF3γ protein levels resulted in 1.193, 0.562 and 0.558 unit increases in NOX4 protein levels in CC, CT and TT genotypes, respectively. 

### Relationship between energy intake per day and *HNF3γ* gene expression in PBMC

As shown in Figure 1E and 1F, a significant negative correlation between energy intake per day and *HNF3γ* mRNA levels was observed for all three genotypes (*r*=0.207, 0.211 and 0.241 for CC, CT and TT genotypes, respectively; all *P*<0.05). The slopes of the three genotypes showed no significant differences (*P*=0.516) for genotype and *HNF3γ* mRNA level interaction. A significant negative correlation was observed between energy intake per day and HNF3γ protein levels in all three genotypes (*r*=0.422, 0.398 and 0.372 for CC, CT and TT genotypes, respectively; all *P*<0.05). No significant differences were observed in the slopes of the three genotypes (*P*=0.740) for genotype and HNF3γ protein level interaction.

### Effects of rs1836882 on affinity between *HNF3γ* and *NOX4* gene promoter region

**Figure 2:**
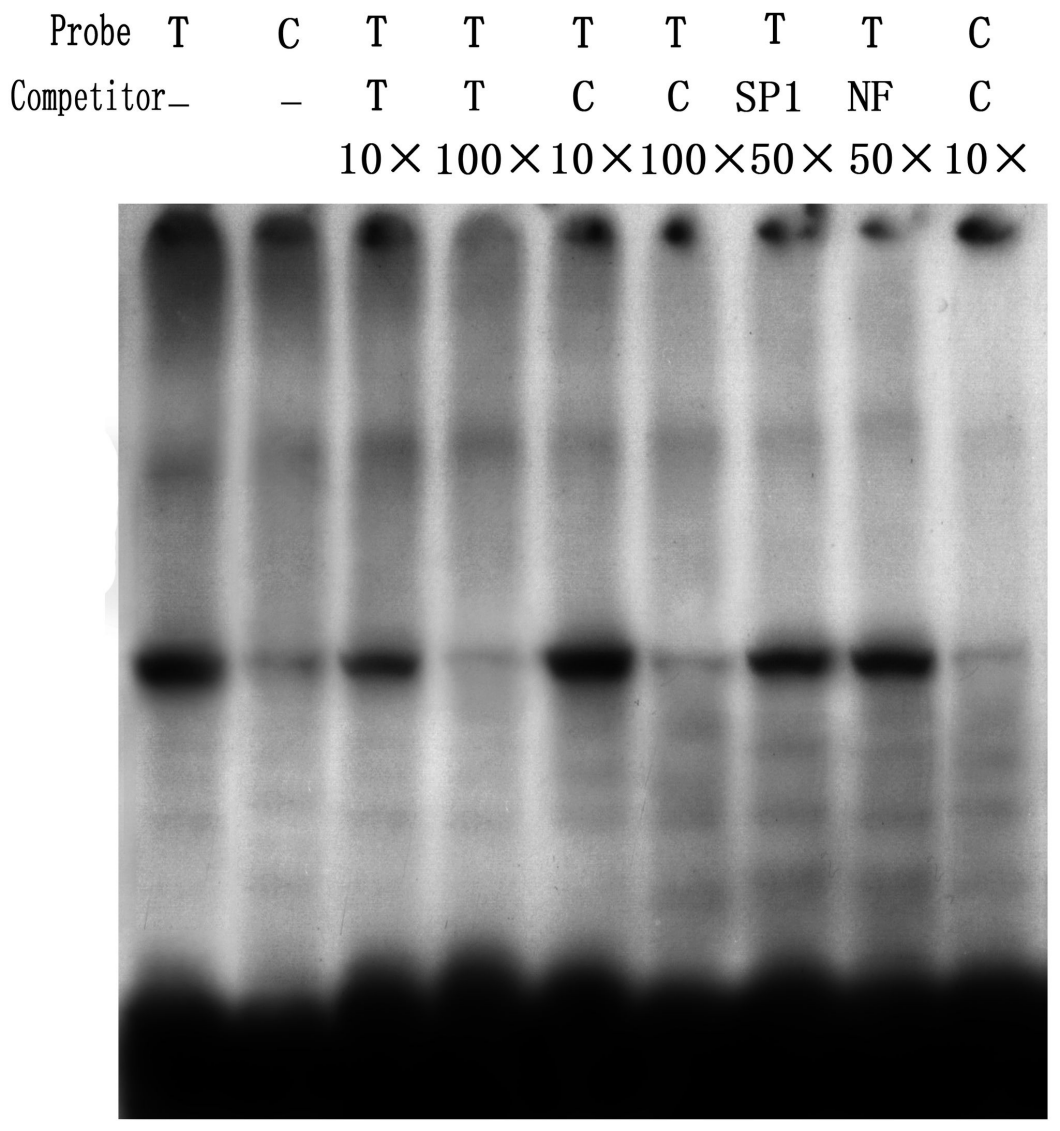
Results of an electrophoretic mobility shift assay for SNP of rs1836882 (C/T). Results show probes with rs1836882 (T) had higher affinity for nuclear protein than probes with rs1836882 (C).

In an electrophoretic mobility shift assay (EMSA) of nuclear extracts from PBMC using double-stranded oligonucleotide probes corresponding to HNF3γ, probes with T produced a speciﬁc band with a higher affinity than that produced by probes with C. Bands disappeared in the presence of a non-labeled T probe as a competitor, but not in the presence of a non-labeled C probe (Figure 2), or in the presence of transcription factors SP-1 or NF-kB.

### Effects of rs1836882 on transcriptional activity of *NOX4* gene

**Figure 3:**
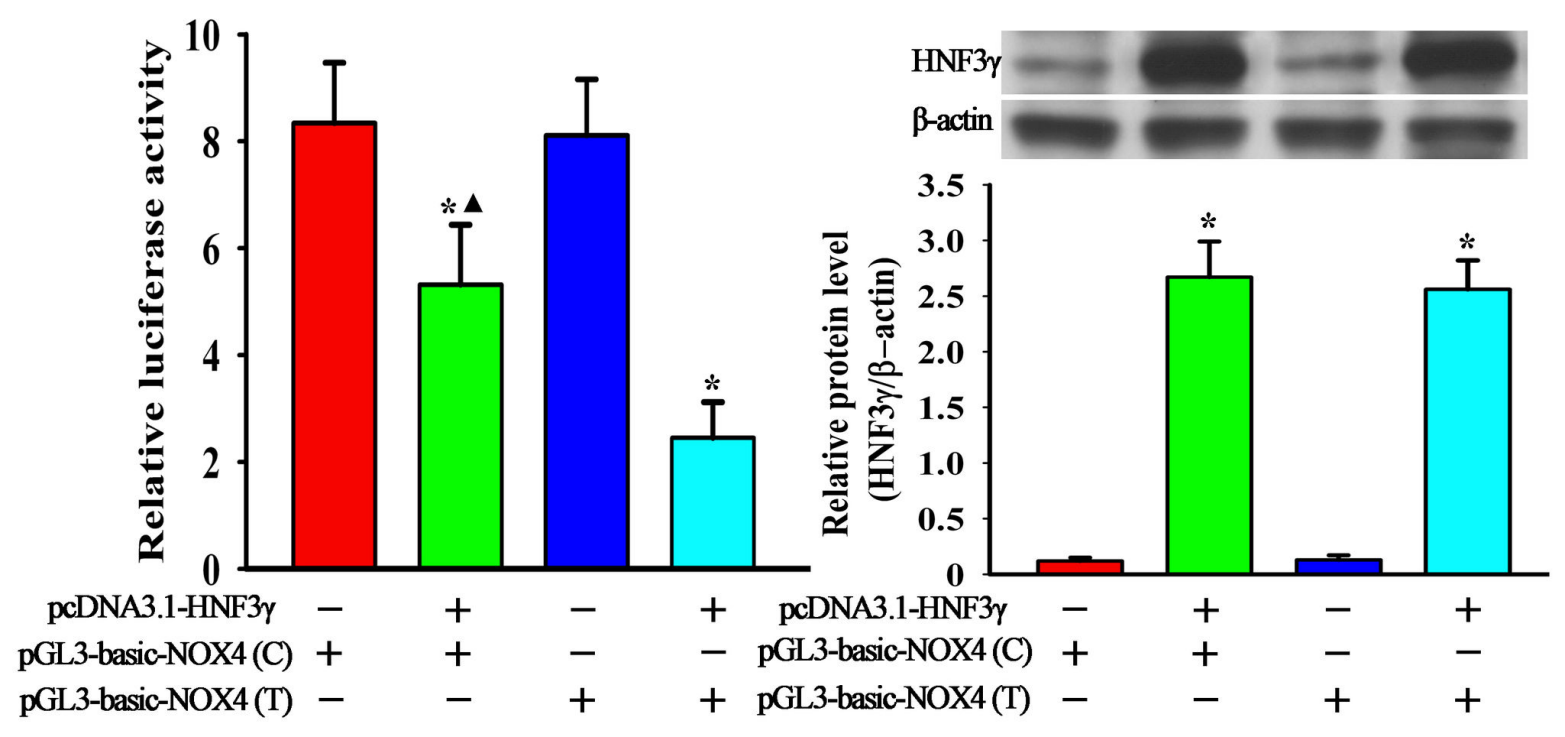
Comparison of relative luciferase activity between pGL3-basic-NOX4 (C) and pGL3-basic-NOX4 (T) plasmids. PBMC were transfected with C- or T-bearing reporter vectors with or without pretreatment of over-expression HNF3γ plasmid. Left panel: Bar chart of alterations in relative luciferase activity. Right panel: Representative western blot (top) and bar chart (below) of alterations in HNF3γ protein levels either pretreated with over-expression HNF3γ plasmid or not. Renilla luciferase activity was measured and normalized to firefly luciferase. Data represent mean ± SD of at least three independent experiments. *P<0.05 *vs*. cells treated with either pGL3-basic-NOX4 (C) or pGL3-basic-NOX4 (T); ▲ *P*<0.05 *vs*. cells treated with over-expression HNF3γ plasmid plus pGL3-basic-NOX4 (T).

To confirm the characteristics of rs1836882 regulating *NOX4* gene transcription, upstream promoter sequences (including rs1836882 of *NOX4* gene) were amplified, an over-expression *HNF3γ* gene plasmid was constructed, and promoter activity was assessed using a luciferase reporter assay. The results suggest that pGL3-basic-NOX4 (T) reporter showed lower, but not significant, transcriptional activity *vs.* that of pGL3-basic-NOX4 (C). Over-expression of HNF3γ significantly decreased pGL3-basic-NOX4 (T) transcriptional activity compared with pGL3-basic-NOX4 (C) activity (5.68% *vs.* 2.12%, *P*<0.05) (Figure 3).

### ROS levels in PBMC induced by serum from high energy intake participants via *HNF3γ*-*NOX4* pathway

**Figure 4:**
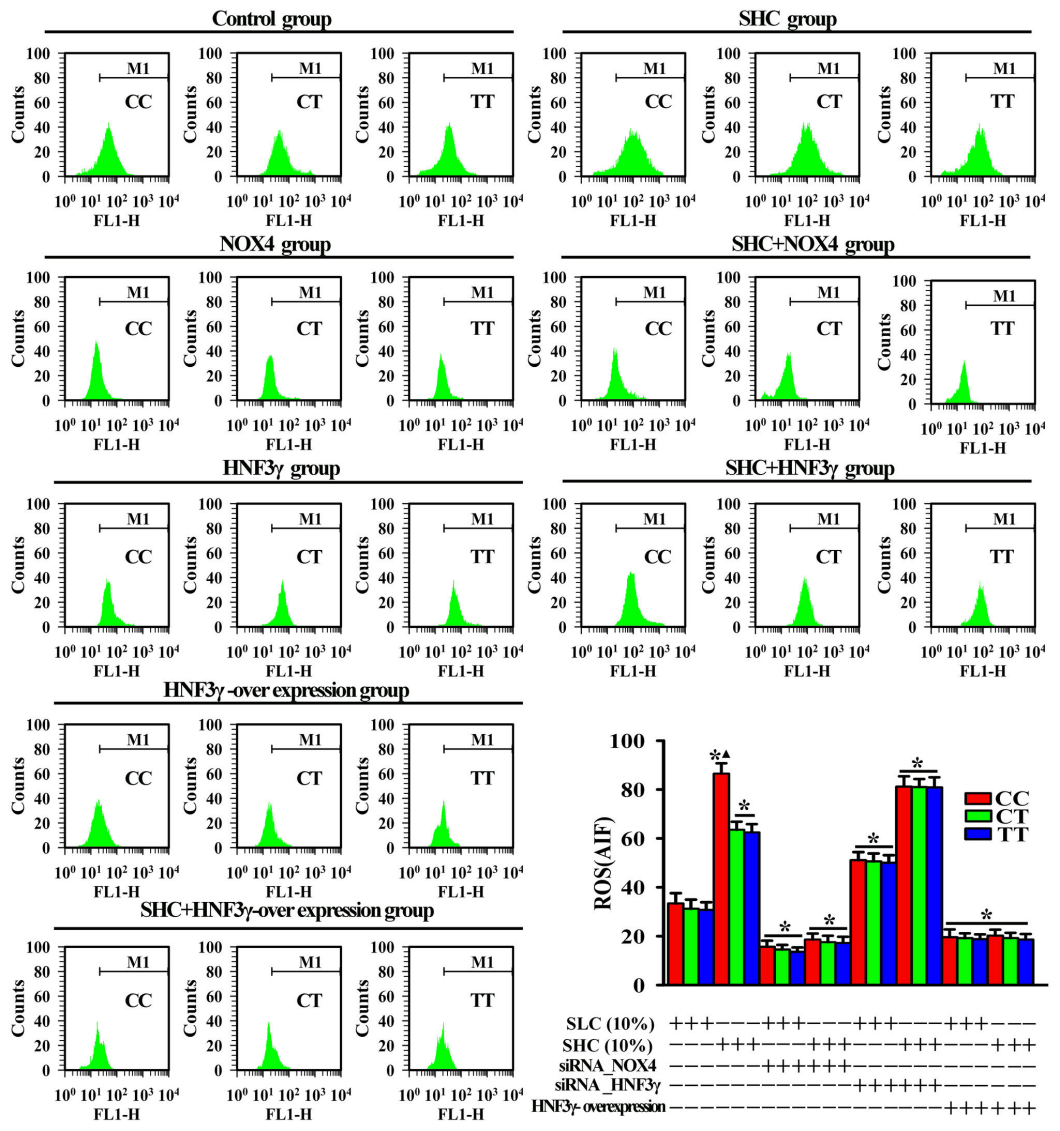
Effects of rs1836882 on ROS levels in PBMC treated with different caloric intake subjects’ serum. Production of ROS was monitored using a DCFH-DA, and average fluorescence intensity of 10,000 cells was analyzed using flow cytometry. There were three replicate wells per treatment and the experiment was carried out three times. Data are expressed as mean ± SD. Lower right corner graph: Genotype-dependent changes in levels of ROS in PBMC after different treatments. * *P*<0.05 *vs*. Control group; ▲ *P*<0.05 *vs*. the other two genotypes within the same group. PBMC: peripheral blood mononuclear cells; AIF: average fluorescence intensity; ROS: reactive oxygen species; DCFH-DA: 2’,7’-dichlorofluorescein diacetate; SHC: serum from high caloric intake group; and SLC: serum from low caloric intake group.

To investigate the potential effects of serum from high caloric intake participants (SHC) on ROS levels in PBMC via the HNF3γ-NOX4 pathway, the levels of oxidative stress in PBMC were evaluated by ﬂow cytometry. In the Control group, although ROS levels showed no significant differences among the three genotypes, there was a tendency for the ROS level of the CC genotype to be higher than the levels of the other two genotypes (CT and TT). Treatment with SHC induced significantly higher ROS levels *vs.* treatment with serum from low caloric intake participants (SLC) in all three genotypes. ROS levels induced by SHC of the CC genotype were significantly higher than that of the CT or TT genotypes within the SHC group. Compared with the Control group, the HNF3γ group had significantly higher ROS levels, while the NOX4 group and the HNF3γ over-expression group had significantly lower ROS levels for all three genotypes. ROS levels showed no significant differences among the three genotypes within the HNF3γ, NOX4 and HNF3γ over-expression groups. ROS levels in the SHC+NOX4 group and the SHC+HNF3γ over-expression group were significantly lower than that of the SHC group for all three genotypes; ROS levels in those two groups showed no significant differences among the three genotypes. ROS levels in the SHC+HNF3γ group was significantly higher than that of the Control group for all three genotypes; there was no significant difference among the three genotypes within the group (Figure 4). (For details on grouping see the Materials and Methods section).

**Figure 5:**
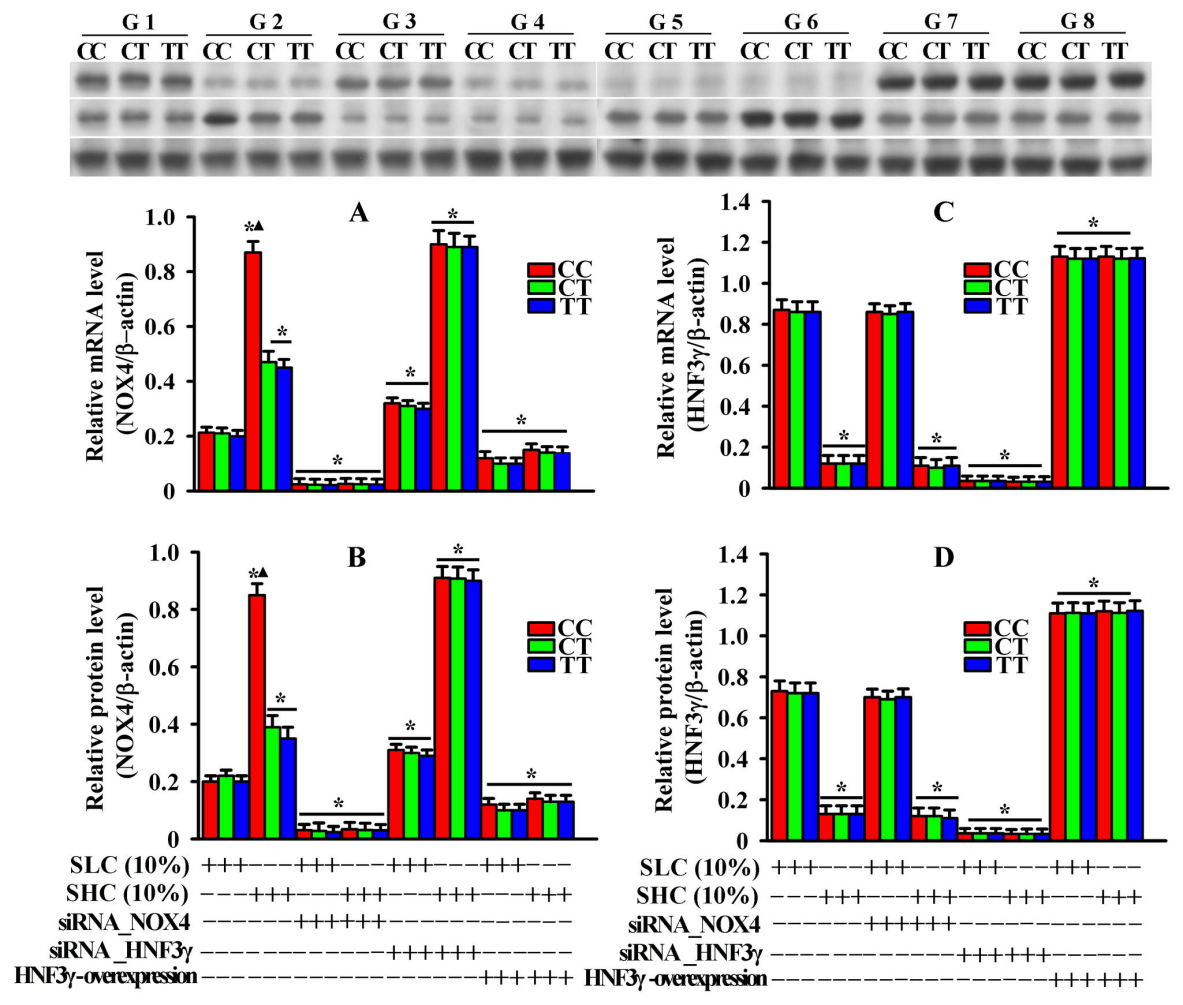
Effects of rs1836882 on NOX4 and *HNF3γ* expression in PBMC treated with different serums. Upper panel: Representative western blots of alterations in NOX4, HNF3γ and β-actin protein levels in PBMC after different treatments. G1: Control group; G2: SHC group; G3: NOX4 group; G4: SHC+NOX4 group; G5: HNF3γ group; **G6**: SHC+ HNF3γ group; **G7**: HNF3γ over-expression group; and **G8**: SHC+ HNF3γ over-expression group. **A** and **B**: Genotype-dependent changes of NOX4 gene mRNA and protein levels, respectively. **C** and **D**: Genotype-dependent changes of *HNF3γ* gene mRNA and protein levels, respectively. * *P*<0.05 *vs*. control group; ▲ *P*<0.05 *vs*. the other two genotypes within the same group. PBMC: peripheral blood mononuclear cells; SHC: serum from high caloric intake group; SLC: serum from low caloric intake group.

*NOX4* gene mRNA and protein levels were significantly up-regulated by SHC, and expression levels of the CC genotype were significantly higher than that of the CT or TT genotype. *NOX4* gene up-regulation induced by SHC was markedly inhibited by over-expression of HNF3γ or siRNA targeting the *NOX4* gene for all three genotypes. Contrary to the *NOX4* gene, mRNA and protein levels of the *HNF3γ* gene were significantly down-regulated by SHC *vs.* the Control group for all three genotypes; there were no significant differences among the three genotypes within the group (Figure 5).

## Discussion

In this study, *HNF3γ* gene expression in PBMC was found to be sensitive to alterations in dietary caloric intake; a finding consistent with other research. The *HNF3γ* gene is classified as part of the Foxa transcription factor family. This family was found to regulate glucagon production and glucose homeostasis, particularly in response to fasting [42]. The Foxa family was also reported to be involved in the regulation of diet-restriction-mediated longevity in *Caenorhabditis elegans* [27]. *FOXA2* (Entrez Gene: 3170) is reported to negatively regulate basal transcription and expression of the human fat mass and obesity-associated gene, which is involved in regulating dietary intake and energy expenditure [43]. In our previous study, we observed that caloric restriction decreased ROS levels in endothelial cells through the promotion of HNF3γ binding to the *NOX4* promoter region and inhibiting *NOX4* gene expression [28]. The aim of the present study was to determine whether rs1836882 (C/T) polymorphism in the *NOX4* gene promoter region—a polymorphism near the binding site of HNF3γ to the *NOX4* promoter region—is able to modify associations between dietary caloric intake and ROS levels in PBMC.

In the population study, *NOX4* gene expression and ROS levels were positively correlated with dietary caloric intake with significant differences in slopes among the three rs1836882 genotypes. No differences were observed in slopes from regression analysis between dietary caloric intake and *HNF3γ* expression, or in slopes from regression analysis between *NOX4* expression and ROS levels among the three genotypes. With the same dietary caloric intake increase, the CC genotype was observed to have the maximum *NOX4* gene expression, maximum ROS levels and minimum *HNF3γ* expression among the three genotypes. 

The *NOX4* promoter region with rs1836882 T genotype showed high binding affinity for HNF3γ protein in EMSA. Luciferase reporter assay showed that the TT genotype promoter region had the lowest transcriptional activity with HNF3γ over-expression. Taking together, these results suggest that the *NOX4* promoter region with the TT genotype had higher binding affinity for HNF3γ and lower transcriptional activity than the other two genotypes. Transcriptional activity heterogeneity could be partly responsible for the different slopes observed with regression analysis between *HNF3γ* and *NOX4* gene expression. Different slopes from negative regression between *HNF3γ* and *NOX4* gene expression among the three genotypes could give rise to modulation of rs1836882 polymorphism associations between ROS levels in PBMC and dietary caloric intake.

According to results from our previous study, there were four HNF3γ binding sites (-6 bp, -249 bp, -76 bp and -954 bp) in the *NOX4* promoter region. When HNF3γ binds to one or more of those four sites, *NOX4* gene expression is repressed sharply [28]. The rs1836882 polymorphism selected for examination in the current study lies near the -954 bp and -249 bp HNF3γ binding sites. The heterogeneous binding ability of the *NOX4* promoter region to HNF3γ may be explained by the T to C mutation in rs1836882 near -954 bp and -249 bp HNF3γ binding sites diminishing adhesion of HNF3γ to the -954 bp and -249 bp sites and consequently attenuating a negative regulatory effect of HNF3γ on *NOX4* expression. This explanation is supported by a recent study conducted by Li et al.. In their study, a T to C base mutation near *FOXA2* binding sites in *FGL1* (Entrez Gene: 2267), *BTG1* (Entrez Gene: 694) and *SERPINA5* (Entrez Gene: 5104) genes was found to decrease *FOXA2* binding to regulating regions of those target genes, and consequently suppressing those genes’ expression [44].

To confirm the effects of dietary caloric intake on *HNF3γ* and *NOX4* expression and ROS levels, an *in vitro* PBMC culture was performed. Consistent with data from the population study, serum obtained from high dietary caloric intake participants induced the highest ROS levels, with maximum *NOX4* and minimum *HNF3γ* expression in the CC genotype. It has been reported that a low calorie diet reduced oxidative stress and inflammatory-related genes, including NADH-coenzyme Q reductase in PBMC, but *NOX4* and *HNF3γ* were not found to be affected by the low calorie diet [29]. This discrepancy may be explained by ethnic differences. In our study, healthy Han Chinese people were recruited, but in the above-mentioned study, Caucasian obese men were recruited. Different ethnicities and baseline weights will affect final results. 

In addition, *NOX4* knockdown by siRNA and over-expression of *HNF3γ* impaired ROS levels increases induced by serum from high dietary caloric intake participants regardless of genotype. This result confirmed the finding from the population study that rs1836882 polymorphism modulates associations between ROS levels in PBMC and dietary caloric intake by affecting HNF3γ binding to the *NOX4* regulating region. But in our study, pretreatment by siRNA targeting *HNF3γ* induced lower *NOX4* expression and ROS levels in PBMC than treatment with serum from high dietary caloric intake participants. This indicates that there remains another mechanism other than *HNF3γ* for high caloric intake increasing *NOX4* expression and ROS levels. 

In a recent study, hypercholesterolemia increased *NOX4* expression and the consequent oxidative stress in the heart by down-regulating *microRNA-25* (Entrez Gene: 407014) [45]. *NOX4* expression and oxidative stress were also down-regulated by Janus-tyrosine-kinase 2 (Entrez Gene: 3716) inhibition [46]. These reports suggest that, apart from *HNF3γ*, *NOX4* expression and the consequent oxidative stress can be regulated by several pathways during diet treatment.

In the current study, we observed that rs1836882 polymorphism in the promoter region of *NOX4* could be a factor in inter-individual differences in associations between dietary caloric intake and ROS levels in PBMC. We observed that the same increase in dietary caloric intake was able to induce the greatest decrease in *HNF3γ* expression, the greatest increase in *NOX4* expression and the greatest increase in ROS levels in PBMC in participants who carried the CC genotype in rs1836882. However, long-term longitudinal study studies are needed to examine the relationship between ROS levels levels in PBMC and clinical events associated with oxidative stress, such as atherosclerosis and myocardium fibrosis, in these participants. With a more complete understanding of this relationship, our results could provide good evidence for the tailoring of caloric restriction programs to individuals on the basis of their genetic patterns.

This study had several limitations. First, dietary caloric intake was based on semi-quantitative self-reported questionnaires, which may allow for the introduction of inaccurate data. However, the questionnaire used included 64 items and was specifically designed for Chinese, and has been shown to have high reproducibility and reliability in Chinese population studies [39]. Also, trained dietitians were included in the research to ensure the validity of the questionnaire from data collection to processing. Second, because of the small sample size, the genetic analysis results should be interpreted with caution. Finally, the 656 participants enrolled were all Han Chinese people. Although other people, such as Europeans, also have rs1836882 polymorphism in the promoter region of *NOX4*, the results from our study should be interpreted carefully considering the heterogeneity of the population that participated.

In summary, the rs1836882 polymorphism in the promoter region of *NOX4* gene modulates associations between dietary caloric intake and ROS levels in PBMC by affecting HNF3γ binding to the *NOX4* promoter region in a healthy Han Chinese population. With the same dietary caloric intake, participants with the CC genotype in rs1836882 showed the highest levels of ROS levels in PBMC. We previously reported caloric restriction decreased ROS levels in endothelial cells through HNF3γ down-regulating *NOX4* gene expression [28]. Taken together, the findings suggest that genetic variations affecting ROS levels in cells interacting between blood and the entire body may explain inter-individual differences in oxidative stress and disease risk in response to over-caloric intake.

## Supporting Information

Text S1 (DOC)
