# Supplementary material for: Polymorphism of rs1836882 in NOX4 Gene Modifies Associations between Dietary Caloric Intake and ROS Levels in Peripheral Blood Mononuclear Cells
Source: PLoS One. 2013 Dec 31;8(12):e85660. doi: 10.1371/journal.pone.0085660 (PMC3877383; doi:10.1371/journal.pone.0085660)
Supplement: Text S1 — (DOC) [file pone.0085660.s001.doc]

The supplementary materials for details of experimental methods

### NOX4 promoter region sequencing

The primers for the promoter regions (-2000bp to +100bp) of NOX4 genes included forward (F: **5′-TCTCATTTGTAGAAACATGGTCAATGTC-3′), reverse (R: 5′-TAACAAACTTATAGGCACAGCACAGAAG-3**′). The length of PCR is 2354bp. Initial denaturation at 95°C for 5 minutes was followed by 35 cycles comprising 95°C for 30 seconds, 65°C for 30 seconds, and 72°C for 3 minutes. The genotypes of product were determined by direct sequencing.

### Electrophoretic mobility shift assay

Peripheral blood mononuclear cells (PBMCs) cells were washed with ice-cold phosphate-buffered saline before suspension in an ice-cold hypoosmotic buffer (10mM HEPES, 2mM MgCl_2_, 0.1mM EDTA, 10mM KCl, 1mM DTT, 1mM PMSF, 1mM NaVO_3_, 10mM NaF, and 1× protease inhibitors). Cells were incubated on ice for 10min, 0.2% Nonidet P-40 was added, and the cells were incubated again for 15min. After centrifugation at 15000g for 30s, the pellets were suspended in cold saline buffer containing 50mM HEPES, 50mM KCl, 300mM NaCl, 0.1mM EDTA, 10% glycerol, 1mM DTT, 1mM PMSF, 1mM NaVO_3_, 10mM NaF, and 1× protease inhibitor. The mixture was incubated on ice for 30min. After centrifugation at 15000 g for 30min at 4℃, the supernatants containing the nuclear proteins were collected. The protein concentrations were determined using a bicinchoninic acid-based protein assay kit (Pierce, Rockford, IL, USA). Nuclear protein aliquots (5 mg) were stored at -80℃ until use. Double-stranded oligonucleotides for NOX4 and the non-speciﬁc competitor SP-1 and NF-kB were used as probes for EMSA. The double-stranded oligonucleotides were annealed at 95℃ for 5min and gradually cooled at room temperature. The double-stranded DNA probes were biotin-labeled at the 3′ position using Biotin 3' End DNA Labeling Kit (Pierce). A 10 mL reaction mixture comprising nuclear protein (5 mg), 75.2 mM NaCl, 20 mM HEPES, 1mM EDTA, 5% glycerol, 0.0504 mM MgCl_2_, 5mM DTT, 1mM NaVO_3_, 10 mM NaF, 400 ng of BSA, and 250 ng of poly (dI-dC) was pre-incubated on ice for 20min. Ten nanograms of biotin-labeled was added, and the reaction was incubated at 15°C for another 15 min. A competition experiment was performed using unlabelled, blunt-ended competitor oligonucleotides added to the binding reaction before the addition of biotin-labeled oligonucleotide. The reaction mixtures were separated by 6.0% non-denaturing polyacrylamide gel electrophoresis at 120 V in Tris-borate-EDTA (TBE) buffer and electrotransferred to a nylon membrane at 300 mA for 30 min. The locations of protein/DNA complexes were visualized by incubation using horseradish peroxidase-conjugatedstreptavidin and an enhanced chemiluminescence kit (Pierce). Nucleotide sequences of oligonucleotides used as probes and competitors are: **SP1 5′-ATTCGATCGGGGGGGGGCGAGC -3′; NF-kB 5′-AGTTGAGGGGACTTTCCCAGG-3′; rs1836882 (C) 5′-GAAGCACTACAGCATAAGGCTTGCG-3′ and rs1836882 (C) 5′-GAAGCACTACAGTATAAGGCTTGCG -3′.**

### Construction of plasmids

A 940 bp fragment of the human NOX4 gene regulation region was prepared by PCR ampliﬁcation using either rs1836882(C) homozygous or rs1836882 (T) homozygous human genomic DNA as a template (forward primer, **5′-GCCGAGCTCCTGAACTTCTTCTGTAACAAGTTC-3′** and reverse primer, **5′-TGAAGATCTGCTATAAAGTTGAATTGCTGAAAC-3′**. The 5′ end of the forward primer carries the SacI enzyme cutting site and that of reverse primer carries the BglII enzyme cutting site. Each PCR product was gel-puriﬁed using an agarose gel puriﬁcation kit (Invitrogen, Carlsbad, CA, USA) and ligated into TA vector (Invitrogen). The plasmid was digested with SacI and BglII (Takara, Shuzo, Japan) and ligated into a similarly digested pGL3-basic luciferase reporter vector (Promega, Madison, WI, USA) using T4 DNA ligase (Takara). The 1053bp full-length HNF3γ CDS was amplified by PCR using genomic DNA from a healthy subject as a template ((forward primer, **5′-CGAATTCGAGAGATCCAGAGCGCTCCGTTC-3′** and reverse primer, **5′-GCTCTAGACAGCCATACCCACCACCATGTTC-3′**). The 5′ end of the forward primer carries the EcoRI enzyme cutting site and that of reverse primer carries the Xbal enzyme cutting site.The expression plasmid (pcDNA3.1-HNF3γ) was obtained by subcloning full-length HNF3γ CDS into the EcoRI and Xbal (Takara) sites of a pcDNA3.1 expression vector (Invitrogen). All constructs were veriﬁed by direct sequencing. Plasmid DNAs were prepared from these constructs using an Endo Free Plasmid Maxi kit (Qiagen, Hilden, Germany). Plasmid DNA concentrations and purity were assessed using UV spectrophotometry and agarose gel electrophoresis.

**Transfection of NOX4 and HNF3γ siRNA for PBMCs**

We used commercially generated double-strand RNA pair oligonucleotides, duplexed Stealth™ RNAi (Invitrogen, Carlsbad, CA, USA), for RNA interference. HNF3γ specific siRNA oligos against the human sequence were as follows: **5′-GAGCCCAGAUUCCAAGCUAUU-3′** and **5′-UAGCUUGGAAUCUGGGCUCUU-3′**. NOX4 specific siRNA oligos against the human sequence were as follows: **5′-GAGCCCAGAUUCCAAGCUAUU-3′** and **5′-UAGCUUGGAAUCUGGGCUCUU-3′**. Stealth™ RNAi Negative Control Low GC Duplex (Invitrogen) was used as the negative control. siRNA was transfected to peripheral blood mononuclear cells (PBMCs) with Lipofectamine 2000 (Invitrogen) according to the manufacturer's instructions. To determine the optimal concentration and time point of siRNA efﬁcacy, PBMCs were grown to subconﬂuency and transfected with 10, 30, or 100 nmol/L of each of the siRNAs in the presence of 8 mmol/L lipofectin and subjected to analysis 24, 36, 48 and 72 hours after transfection. Transfected cells were used for further experiments.

### Real-time RT-PCR analysis

Samples were amplified in the Applied Biosystems 7900 HT Fast Real-Time PCR System (Applied Biosystems, Foster City, CA, USA). The primers for NOX4 mRNA included forward (F: **5′-TCACCGTTGGAACTACACTGTGTG-3′, reverse (R: 5′-TATTTTTAGTAGCGATGGGGTTTC-3′**). The primers for HNF3γ mRNA included forward: (F: **5′-CGGGCAAGATGCTGACCTTGAGTG-3′, reverse (R: 5′-** **GTAGGAGCCCTTGCCAGGCTTGTC -3′**).
